# Supplementary material for: Identification and Characterization of Development-Related microRNAs in the Red Flour Beetle, Tribolium castaneum
Source: Int J Mol Sci. 2023 Apr 3;24(7):6685. doi: 10.3390/ijms24076685 (PMC10094939; doi:10.3390/ijms24076685)
Supplement: Supplementary file 1 [file ijms-24-06685-s001.zip › Figures.pdf]

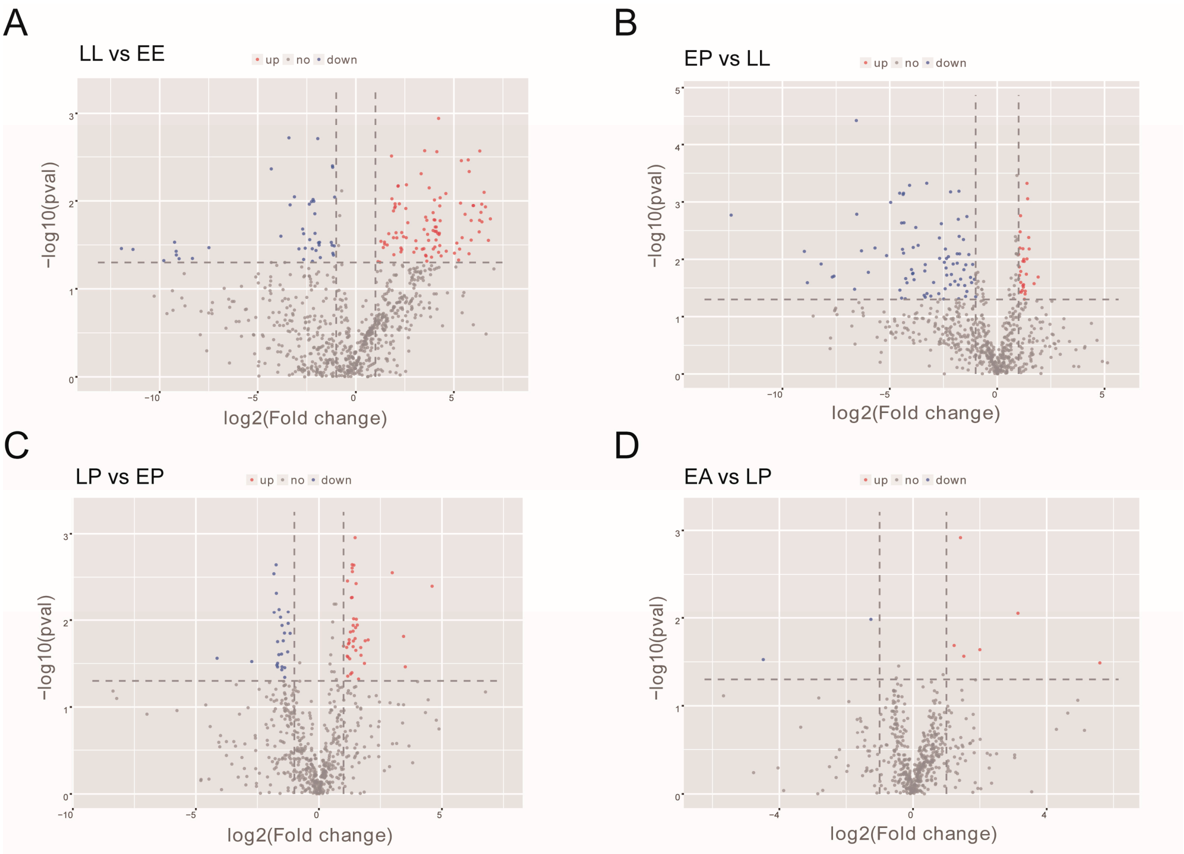

Figure S1. Volcano plots of miRNAs in the pairwise LL vs. EE (A), EP vs. LL (B), LP vs. EP (C), and EA vs. LP (D). Red points means upregulated miRNAs ($p < 0.05$), blue points means downregulated miRNAs ($p < 0.05$), and grey dots indicate miRNAs with no difference in expression between two developmental stages.

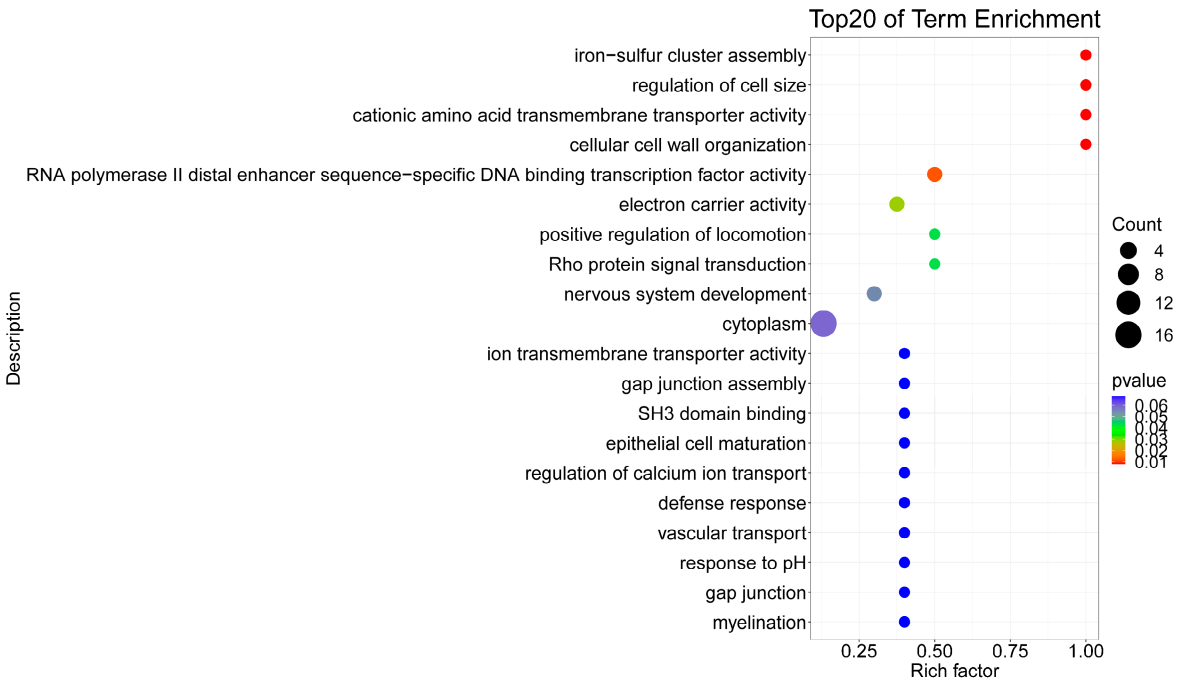

Figure S2. GO enrichment analysis of cluster E DEmiRNA targets. The *Y*-axis indicates the GO term, and the *X*-axis represents the rich factor.
